# Developing Micro/Nanostructured Fluidic Mixing Technology for Biomedical Applications

**DOI:** 10.1002/advs.75562

**Published:** 2026-05-07

**Authors:** Junkai Wang, Hanxu Chen, Yile Fang, Yuanjin Zhao

**Affiliations:** ^1^ Department of Rheumatology and Immunology School of Biological Science and Medical Engineering Nanjing Drum Tower Hospital Southeast University Nanjing China; ^2^ Wenzhou Institute University of Chinese Academy of Sciences Wenzhou Zhejiang China

**Keywords:** biomedical applications, fluidic mixing, hydrodynamics, micro/nanostructured, microfluidics

## Abstract

Fluid mixing technologies are indispensable in the fields of biomedicine and biotechnology, where precise control over mixing processes is essential for optimal performance. Traditional mixing systems, such as stirred tanks, jet‐flow mixers, and mechanical drives, often suffer from limitations in terms of precision, scalability, and energy efficiency, especially when adapted to complex biomedical applications. Recent developments in micro/nanostructured fluid mixing technologies offer promising solutions to these challenges by enabling efficient, controllable, and energy‐saving mixing at the microscale. These technologies leverage intricate micro/nanostructures that can enhance diffusion, induce vortices, and generate chaotic convection, thus overcoming the constraints faced by conventional methods. In this paper, we systematically classify these micro/nanostructures based on the fluid mixing phenomena they exploit and review their applications across a range of biomedical fields, including biomaterials fabrication, drug development, cell culture, organs‐on‐chips, biosensing, and diagnostics. We also examine how these technologies contribute to advancements in precision medicine, personalized treatment, and high‐throughput testing. Finally, we discuss the future challenges, opportunities, and interdisciplinary collaboration needed to further advance the clinical and industrial adoption of micro/nanostructured fluid mixing technologies.

## Graphical abstract

This review critically evaluates how micro/nanostructured mixing technologies are redefining biomedical research. By synergizing fundamental analysis, numerical modeling, structural design, and external field manipulation, these systems attain unprecedented control over mass transport. We highlight their transformative impact on biomaterials, drug development, organs‐on‐chips, and diagnostics, offering a strategic roadmap for future clinical and industrial translation.

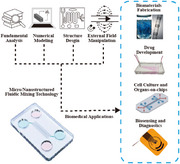

## 1 Introduction

In the rapidly expanding field of industrial processes, fluid mixing technologies have emerged as widely adopted and effective tools. Complex driving system (e.g., stirred tanks [1], jet‐flow high‐shear mixers [2], static mixers [3], and ultrasonic homogenizers [4]) concentrates energy to disperse fluids into droplets or homogenized phases for mixing [5, 6, 7, 8]. Through efficient mixing, liquid components can achieve molecular‐level dispersion, which helps alleviate system heterogeneity resulting from gradients in phase, viscosity, concentration, or temperature [9]. This technology is widely used across sectors like chemicals, pharmaceuticals, cosmetics, food, and beverages [3, 10, 11, 12]. In such manufacturing processes, different mixing operations can be required, such as miscible fluidic mixing [13], immiscible fluidic dispersion [14], emulsions [15], and miscible blending of non‐Newtonian fluids [16]. To quantitatively evaluate power consumption and elucidate the fluid dynamic mechanisms during the mixing process, computational fluid dynamics (CFD) has been widely used as a modeling and analytical tool [17, 18, 19, 20, 21]. However, conventional macroscale mixing approaches still face significant challenges when handling suspensions containing solid particles with heterogeneous sizes and multi‐phase liquid systems with distinct physical properties [22, 23, 24]. In addition, achieving efficient mass and energy transfer remains challenging because mixing speeds are difficult to standardize across different process configurations. This limitation often results in increased energy consumption and non‐uniform mixing distributions at the macroscopic scale [25, 26, 27, 28, 29, 30]. Non‐optimized design and mixing strategies may fail to achieve the intended performance and may result in substantial resource and energy losses [3]. In biomedical processing, conventional liquid‐mixing approaches, including mechanical stirring, vortex mixing, and bulk ultrasonic treatment, are commonly used for sample preparation, reagent homogenization, and suspension processing. However, these methods often lack precise control over microscale transport phenomena and may introduce sample loss, contamination risk, or excessive energy input. Therefore, novel fluidic mixing strategies for biomedical applications still need further development.

Recently, there has been growing recognition of the absolute necessity of fabricating custom‐designed micro/nanostructures to enhance the fluidic mixing efficiency, especially in the field of biomedical applications. Unlike traditional liquid mixing approaches, advances in biomedical engineering have introduced more accessible and efficient strategies for microscale fluid manipulation, typically on the order of microliters or nanoliters (10^−9^–10^−6^ L), using micro/nanochannels with characteristic dimensions of approximately 10^−9^–10^−6^ m [31]. A series of microfluidic technologies have been developed based on different micro/nanostructures through micro/nano additive manufacturing methods [32, 33, 34] (e.g., two‐photon polymerization [35], stereolithography (SLA) [36], and inkjet printing [37]). Indeed, these micro/nanostructures have major advantages compared to large‐scale systems because of lower power consumption, higher mass transfer efficiency, lower cost, and portability [38, 39, 40]. These structures are capable of inducing secondary flows, shortening diffusion pathways, creating chaotic advection, and generating vortices, thereby effectively enhancing mixing efficiency under different flowing patterns, like laminar flow, turbulence, and vortex [41]. Furthermore, micro/nanostructures can be combined with external driving forces such as acoustic field, magnetic field, or electric field to achieve active and precise control of mixing dynamics. Such designs enable high‐throughput, precise, and energy‐efficient fluid processing. Micro/nanostructured fluidic mixing technology allows precise control over nucleation, growth kinetics, and mass transport during nanomaterial synthesis. Such control enables the production of nanomaterials with narrow size distributions, uniform morphology, and reproducible physicochemical properties, which are critical for biomedical applications such as targeted drug delivery, enhanced biosensing sensitivity, and controlled therapeutic release [31, 42, 43, 44].

**FIGURE 1:**
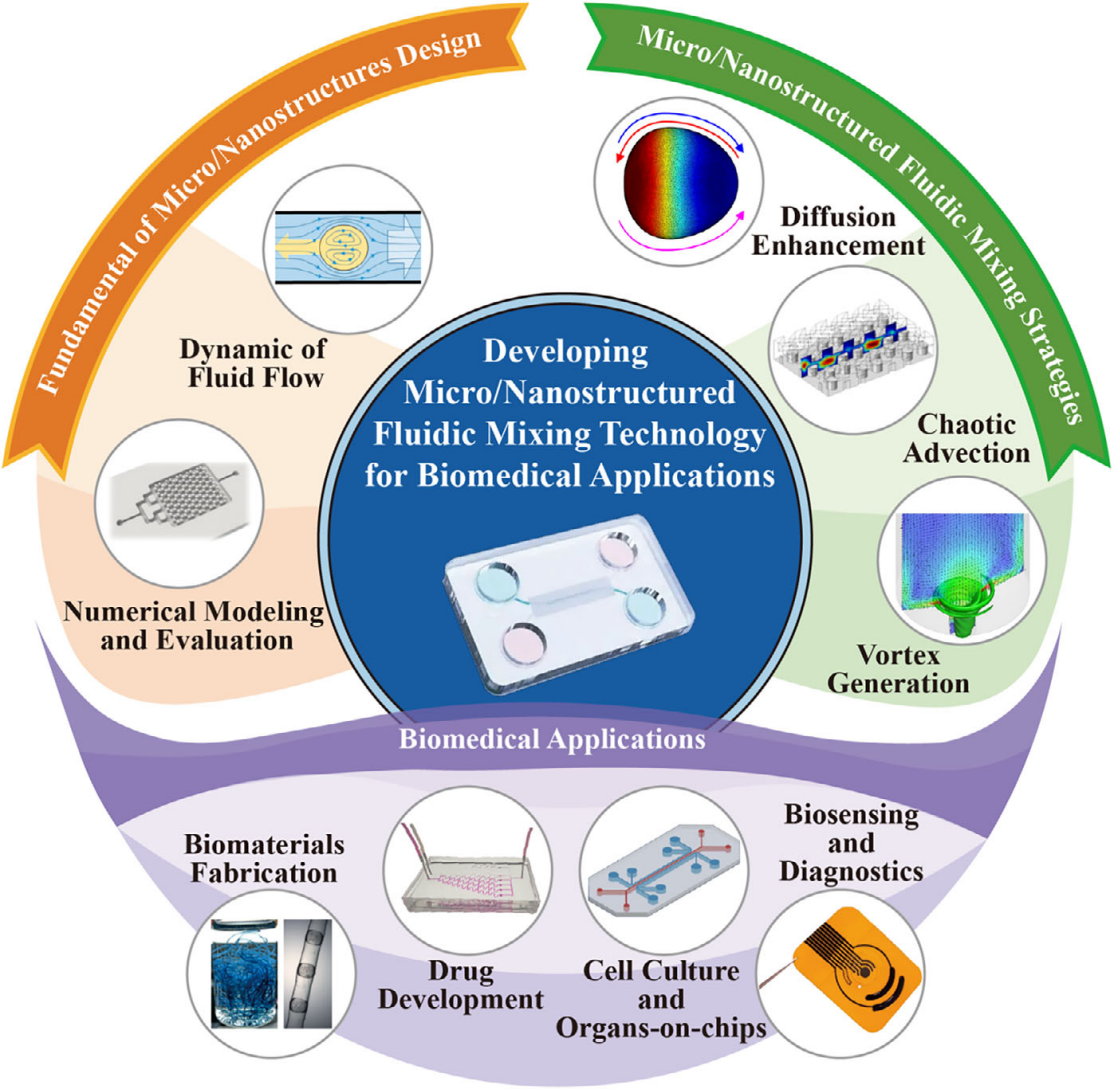
Scheme of micro/nanostructured fluidic mixing technologies for various biomedical applications.

While microfluidic technologies have advanced rapidly, there is still a lack of understanding regarding how design principles for micro/nanostructured mixers dictate both mixing efficiency and biomedical performance. In particular, previous studies have not systematically elucidated the intrinsic relationships among structural geometry, externally applied physical fields for mixing enhancement, and the resulting flow behaviors within micro/nanostructures under diverse application scenarios [45, 46, 47, 48, 49]. In this review, a “structures to physical phenomena to applications” framework is established for micro/nanostructured fluidic mixing technology in biomedical contexts (as illustrated in Figure 1). Distinct from conventional classifications that simply divide mixing strategies into active and passive approaches, the present work categorizes micro/nanostructured fluidic mixing technologies according to the dominant physical mechanisms, including diffusion enhancement, chaotic advection, and vortex flow generation. This classification provides deeper insight into the intensity and effectiveness of different mixing strategies, thereby offering practical guidance for the rational design of micro/nanostructures tailored to specific mixing requirements. Furthermore, representative biomedical applications enabled by these mixing strategies are systematically summarized, encompassing biomaterials fabrication, drug development, cell culture, organs‐on‐chips, biosensing, and diagnostics. These advances collectively demonstrate that micro/nanostructured fluidic mixing technology has become a critical component in modern biomedical engineering. Compared with existing reviews that predominantly focus on device design, this work emphasizes the fundamental role of microscale flow phenomena in bridging structural design and practical implementation. Although emerging efforts have begun to explore the integration of micro/nanostructures with artificial intelligence, the pathway toward fully automated and intelligent systems remains underdeveloped. Future progress will require not only a profound understanding of established studies but also the integration of interdisciplinary approaches and innovative design paradigms. Consequently, the translation of micro/nanostructured fluidic mixing technology into clinically viable solutions represents both a promising opportunity and a significant challenge for the field.

## 2 Fundamentals of Micro/Nanostructure Design

### 2.1 Dynamics of Fluid Flow at the Micro/Nanoscale

In micro/nanostructures, the fundamental dimensionless numbers, flow behavior, and mass transfer characteristics of the fluid together determine the mixing efficiency within the system. Accurately characterizing these factors is of great significance for understanding and optimizing the mixing process at the micro/nanoscale [48].

In microfluidic systems, commonly used dimensionless numbers include the Reynolds number (*Re*) and the Péclet number (*Pe*) [50], which are used to describe the flow state and mass transfer mechanism, respectively. The *Re* is defined as the ratio of inertial to viscous forces and is used to determine whether the flow in micro/nanostructures is laminar or turbulent, given by Equation (1).

(1) $$Re=\frac{Ul}{v}$$

where *U* is the mean flow velocity, *ν* the kinematic viscosity, and *l* is the feature length. If the channel has a circular cross‐section, the pipe diameter is usually selected as the characteristic length; for non‐circular channels, the hydraulic/equivalent diameter, *D_h_*, is commonly used as the characteristic length. As shown in Equation (2).

(2) $$D_{h}=\frac{4A}{P}$$

here, *A* represents the cross‐sectional area of the channel, while *P* denotes its wetted perimeter. It is generally believed that when *Re* ≤ 2320, the viscous force dominates the flow in the pipe, and the liquid in the structure is in a laminar flow state. When *Re* > 2320, the inertial force dominates the flow in the pipe, and the fluid is in a turbulent flow state. The *Pe* is used to represent the relative importance between convective mass transfer and molecular diffusion, and is given by Equation (3).

(3) $$Pe=\frac{UL_{c}}{D}$$

in this context, *L_c_* represents the characteristic mixing length, and *D* represents the mass diffusion coefficient. It is generally accepted that diffusion dominates mass transport when *Pe* ≤ 1, whereas convective transport becomes predominant when *Pe* > 1.

Building upon these dimensionless parameters, the flow and transport behaviors of fluids in micro/nanostructures are governed by the conservation equations of mass, momentum, and energy, which collectively determine their evolution within complex geometries, as expressed in Equations (4)–(6).

(4) $$\frac{\partial (\alpha \rho )}{\partial t}+\nabla \cdot \left(\alpha \rho u\right)=0$$

(5) $$\frac{\partial (\alpha \rho )}{\partial t}+\nabla \cdot \left(\alpha \rho u\right)=-\alpha \rho \nabla p+\nabla \cdot \left(\alpha t\right)+\alpha \rho g+F$$

(6) $$\frac{\partial (\alpha \rho )}{\partial t}+\nabla \cdot [\left(\alpha \left(\rho E+p\right)u\right)=\nabla \cdot \left(\alpha k\nabla T\right)+S_{E}$$

where α defines the volume fraction, *ρ* defines the density, *F* defines the interphase force, *S_E_* defines the energy source term. The sum of the volume fractions of the entire system must be 1 according to Equation (7):

(7) $$\alpha_{1}+\alpha_{2}=1$$

The closure of the momentum equation is achieved by introducing interphase force models to ensure the balance of all forces and momentum conservation, while the closure of the energy equation is accomplished through the energy conservation law to guarantee thermal balance during the fluid mixing process.

Under the coupled effects of flow and mass transfer, the evolution of the concentration field serves as a key indicator for evaluating mixing performance. The degree of mixing uniformity is typically quantified by the standard deviation of concentration distribution, based on which a mixing index (*MI*) is defined, as shown in Equation (8):

(8) $$\mathrm{MI}=\left[1-\sqrt{\frac{1}{N}\sum_{i=1}^{N}{\left(\frac{c_{i}-c_{a}}{\sigma }\right)}^{2}}\right]\times 100\%$$

In the formula, *MI* represents the mixing degree of the fluid in the selected area, *N* represents the number of nodes distributed on the selected cross‐section, *c_i_* is the volume fraction, concentration, or mass fraction of any node within the selected cross‐section, *c_a_* is the ideal concentration value of the volume fraction, concentration, or mass fraction within the selected cross‐section. Based on the above equation, a larger value of *MI* indicates a higher mixing efficiency. *MI* ranges from 0% for an unmixed state to 100% for complete mixing.

In micro/nanochannels, the *Re* is typically low, resulting in stable laminar flow with limited interfacial renewal, whereby mixing is predominantly governed by molecular diffusion [51]. Inducing transverse flow via structural perturbations has become an effective strategy for enhancing mixing efficiency. In curved channels, centrifugal effects generate secondary flows, known as Dean flow [52], whose intensity is commonly characterized by the Dean number (*De*), as given in Equation (9).

(9) De=ReDh2R

where *R* denotes the average radius of curvature of the channel. Increasing the characteristic diameter or decreasing the radius of curvature enhances the intensity of *De*, thereby promoting transverse mixing. However, excessively complex geometries introduce additional energy dissipation, which may compromise the overall flow performance [53]. Previous studies have demonstrated that periodic reconstruction of fluid interfaces can enhance mass transfer while mitigating the suppressive effect of friction on effective *De* [54, 55, 56, 57]. These complexities necessitate systematic evaluation and optimization strategies, for which numerical modeling has emerged as an essential tool to quantitatively analyze flow dynamics and mixing performance in micro/nanostructures.

### 2.2 Numerical Modeling and Evaluation of Micro/Nanostructure

Building upon the fundamental understanding of fluid flow and mass transfer, numerical modeling and evaluation have become essential tools for the design and analysis of micro/nanostructures. These approaches provide new avenues for understanding and optimizing mixing performance by enabling systematic investigation of flow and transport behaviors under controlled conditions.

Numerical modeling has become an essential tool for analyzing fluid flow and mass transport in micro/nanostructured fluidic mixing technology [58]. Built upon a fundamental understanding of transport phenomena, finite element analysis (FEA) provides an effective framework for simulating liquid mixing processes within micro/nanostructures under well‐controlled conditions [48]. Numerical simulations provide deep insights into fluid flow behavior and mass transport mechanisms under various geometrical configurations and operating conditions, while enabling rapid optimization of mixing strategies prior to costly fabrication and experimental validation [59].

A typical finite element modeling workflow involves constructing the geometry of the micro/nanostructured fluidic mixing structure, followed by discretizing the computational domain into finite elements. Appropriate boundary conditions are then applied, including inlet velocity, outlet pressure, and no‐slip wall conditions, after which the coupled governing equations are numerically solved. For most fluids operating within the continuum regime of micro/nanostructures, the mixing behavior is primarily governed by the continuity equation, the Navier‐Stokes equations, and the convection‐diffusion equation, as expressed in Equations (10)–(12).

(10) ∇·u=0

(11) $$\rho \;\left[\frac{\partial u}{\partial t}+\left(u\cdot \nabla \right)u\right]=-\nabla p+\mu \nabla^{2}u$$

(12) $$\frac{\partial c}{\partial t}+\left(u\cdot \nabla \right)c=D\nabla^{2}c$$

In these equations, *u* is the velocity vector, *t* the time variable, *p* the pressure, *µ* the dynamic viscosity and *c* the solute concentration. Finite element analysis primarily focuses on the numerical solution of these governing equations to obtain detailed velocity and concentration fields, thereby providing a solid foundation for evaluating mixing performance in complex micro/nanostructures [48].

Subsequent post‐processing is performed to analyze the velocity and concentration distributions. In particular, the concentration field quantified by the *MI* serves as a key indicator for tracking the volume fraction distribution and flow dynamics within micro/nanostructures. In addition, concentration contour plots are commonly used for qualitative visualization, where a uniform color distribution indicates effective mixing, whereas irregular patterns suggest insufficient mixing [60, 61]. To ensure the reliability of numerical predictions, validation against experimental data, analytical solutions, or previously reported numerical results is required to confirm model accuracy, with acceptable deviations typically within a reasonable error range (about 10%) [62].

FEA has emerged as a powerful tool for the design optimization and performance evaluation of micro/nanostructured fluidic mixing strategies, particularly in providing predictive insights prior to costly fabrication. By integrating numerical simulations with experimental validation, researchers are able to efficiently identify optimal micromixer geometries for applications spanning biomedical engineering, chemical synthesis, and medical diagnostic process [63]. Such simulation‐driven approaches enable systematic exploration of geometric configurations and operating conditions, significantly reducing trial‐and‐error efforts in experimental studies. Although some output parameters inherently rely on the fuzzy logic, they remain robust and cost‐effective tools for predicting and optimizing liquid mixing performance in micro/nanostructured fluidic mixing technology [64].

### 2.3 Evolution of Micro/Nanostructured Fluidic Mixing Technologies

The application of micro/nanostructures in fluidic systems can be traced back to the introduction of the lab‐on‐a‐chip concept in 1979, which was initially developed for gas chromatography systems. This early framework laid the foundation for subsequent advances in microscale fluid manipulation and the development of fluidic mixing technologies [65]. The evolution of micro/nanostructured fluidic mixing strategies was further influenced by the emergence of the micro total analysis system (µTAS) in 1990, which established microfluidic platforms as powerful tools for chemical sensing and analytical applications [66]. At this early stage, fluid transport within microchannels was predominantly governed by laminar flow conditions (*Re* ≪ 1), under which mixing relied almost entirely on molecular diffusion. In 1998, a significant breakthrough was achieved with the introduction of hydrodynamic focusing, which utilized convective effects to compress fluid streams and reduce the characteristic diffusion length. This approach markedly enhanced mixing efficiency and reduced mixing distance, representing a transition from purely diffusion‐dominated mechanisms to convection‐assisted strategies [67].

In 2000, further improvements were realized by extending channel geometries from planar configurations to 3D serpentine structures. These designs induced secondary flows, increased interfacial area, and enhanced mixing efficiency by several orders of magnitude, thereby significantly improving passive mixing performance [68]. In 2002, the incorporation of internal microstructures, such as grooves and surface textures, enabled the generation of chaotic advection within microchannels. These features, readily fabricated using planar lithography techniques, promoted transverse flow and repeated stretching and folding of fluid elements, offering a new paradigm for enhancing mixing under steady pressure‐driven conditions [69]. Between 2000 and 2005, increasing attention was directed toward active mixing strategies. During this period, micromixers were systematically categorized into passive and active types, with the latter employing externally applied fields to improve mixing performance, including acoustic, electric, magnetic, optical, and thermal perturbations. These approaches introduced additional degrees of freedom beyond structural design, enabling more flexible and controllable fluid manipulation. In recent years, growing efforts have focused on synergistic strategies that combine passive structures with active actuation to further enhance mixing efficiency and expand application scope [45, 70]. Since approximately 2005, microfluidic systems have progressively expanded toward biomedical applications. One of the most notable developments was the emergence of organ‐on‐a‐chip platforms, which integrate microfluidic control with biomimetic cellular environments. Over more than a decade of development, various organ models have been successfully established, including lung, heart, intestine, and skin chips. These developments demonstrate significant potential for replacing animal models in drug screening and biomedical research [71].

**FIGURE 2:**
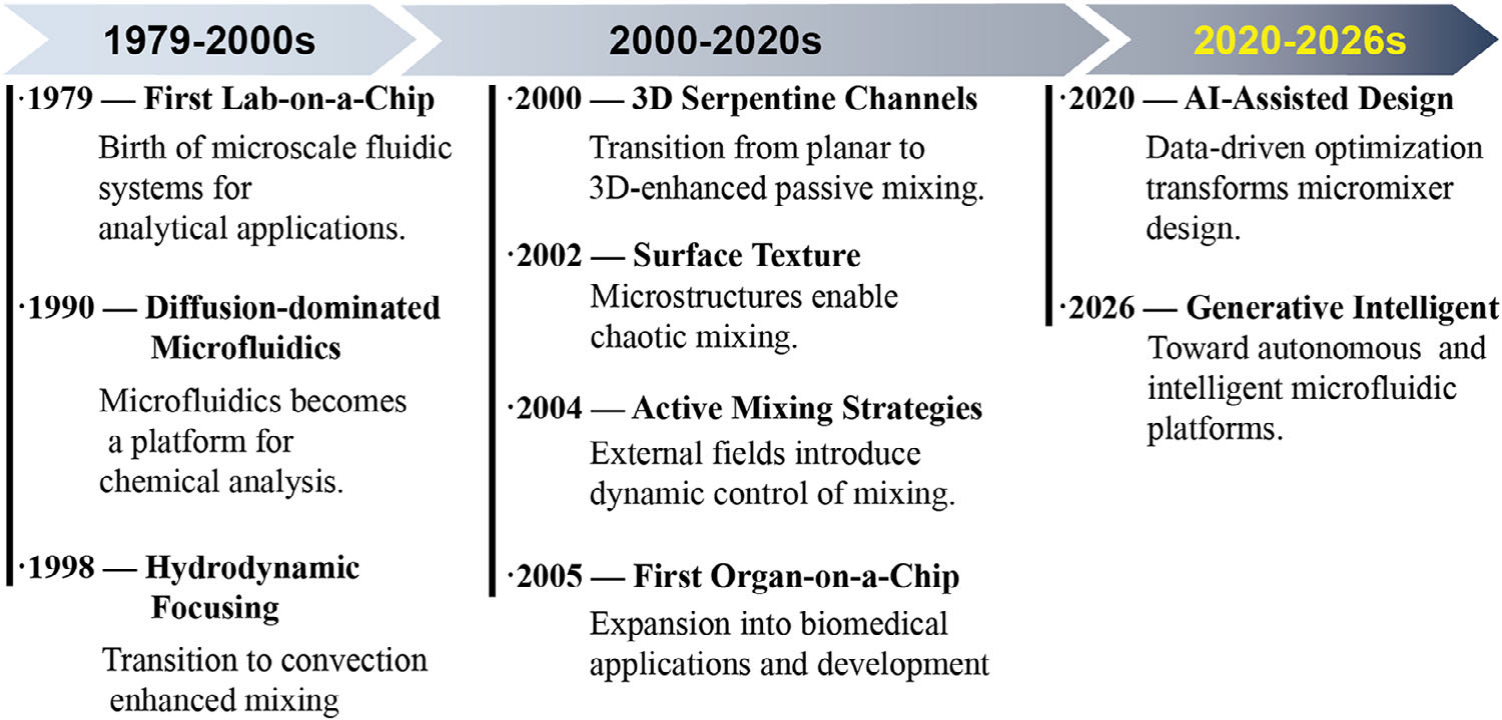
Schematic illustration of the key developmental milestones in micro/nanostructured fluidic mixing technologies.

Since 2020, the rapid advancement of artificial intelligence has introduced a new paradigm for the design of micro/nanostructured fluidic mixing systems. Data‐driven and algorithm‐assisted approaches enable efficient exploration of complex design spaces, facilitating the discovery of high‐performance micro/nanostructures beyond conventional trial‐and‐error methods [72, 73, 74, 75]. Looking ahead, the integration of generative artificial intelligence is expected to further advance intelligent, automated, and system‐level optimized microfluidic platforms, representing a promising direction for improving the efficiency, adaptability, and scalability of micro/nanostructured fluidic mixing technologies [76]. As shown in Figure 2, the critical milestones in the development of micro/nanostructured fluidic mixing technologies are outlined, providing a comprehensive overview from early structural designs to emerging intelligence‐driven approaches. This evolutionary trajectory underscores a shift toward higher efficiency, controllability, and system‐level integration. Looking forward, micro/nanostructured fluidic mixing technologies hold tremendous promise for biomedical applications, offering scalable and adaptable solutions for next‐generation biomedical research.

## 3 Micro/Nanostructured Fluidic Mixing Strategies

The rapid development of micro/nanostructured systems has led to a broad and diverse range of strategies for enhancing fluid mixing, resulting in a substantial body of literature on micromixer design and performance. Existing studies have extensively explored various structural configurations, external actuation methods, and their associated transport characteristics, establishing a solid understanding of the fundamental mechanisms governing microscale mixing. These mixing strategies are generally classified into passive and active approaches. Passive micromixers rely on micro/nanostructure design to manipulate fluid flow and enhance mixing, whereas active micromixers utilize external fields to perturb the flow and improve mixing efficiency. Both approaches have been widely recognized as effective means to enhance interfacial interactions and mass transport [77].

Despite these advances, reported mixing strategies are often presented as isolated micro/nanostructure designs, with limited emphasis on the underlying physical mechanisms that dictate their performance [78]. In particular, limited attention has been devoted to systematically elucidating how fluidic transport phenomena in micro/nanostructures govern fluid mixing behavior, such as the balance between convective transport and molecular diffusion characterized by the *Pe*, as well as the intensity of secondary flows induced by transverse perturbations as reflected by the *De*. To bridge this gap, representative micro/nanostructured designs are systematically organized in Table 1 based on the underlying physical mechanisms driving fluid mixing. This classification unifies both passive and active strategies within a common framework, while further incorporating fabrication complexity and mixing efficiency. Such an approach enables a more systematic comparison across different micro/nanostructures and provides deeper insights into how structural design and external actuation collectively regulate fluid mixing behavior.

**TABLE 1:** Classification of micro/nanostructures based on distinct physical phenomena arising from fluid mixing and their corresponding mixing efficiencies.

Physical Phenomena	Representative Structures	Working Principle	Fabrication complexity	Mixing Efficiency	Ref.
Diffusion Enhancement	– Nanotextured surfaces	– Modifies interfacial morphology and induces local perturbations to enhance mass transport.	High	– Enhanced diffusion at small scales	[79, 80, 81, 82, 83, 84]
	– Thermocapillary	– Generates Marangoni flows due to temperature‐induced surface tension gradients.	High	– Efficient interfacial mixing; temperature‐dependent	[85, 86, 87, 88, 89, 90, 91, 92, 93]
	– Electrothermal	– Induces fluid motion through electric‐field‐driven thermal gradients.	High	– Enhanced convection–diffusion; field‐dependent	[48, 94, 95]
Chaotic Advection	– Split and recombine (SAR)	– Repeatedly splits and recombines fluid streams to increase interfacial area.	Moderate	– Effective via interface stretching	[96, 97, 98, 99, 100, 101, 102]
	– 3D SAR	– Extends SAR structures into three dimensions to enhance flow folding.	High	Improved over planar designs	[16, 103, 104, 105, 106, 107, 108, 109, 110, 111]
	– Asymmetric	– Introduces geometric asymmetry to induce chaotic advection and flow disturbance.	Low to Moderate	– Enhanced interfacial contact	[63, 112, 113, 114, 115, 116, 117, 118]
	– Magnetic actuation	– Applies magnetic fields to induce microscale flow perturbations.	High	– Field‐dependent enhancement	[119, 120, 121, 122, 123]
Vortex Flow Generation	– Contraction‐expansion	– Generates vortex shedding and recirculation through abrupt geometric variations.	Low to Moderate	– Rapid mixing via vortices	[19, 124, 125, 126, 127, 128, 129]
	– Centrifugal lab‐on‐a‐disk (LOD)	– Utilizes rotational forces to induce Dean and Coriolis flows.	Moderate	– Efficient under rotation	[130, 131, 132, 133, 134]
	– Surface acoustic wave (SAW)	– Induces acoustic streaming via high‐frequency surface vibrations.	High	– Fast mixing; power‐dependent	[135, 136, 137, 138, 139, 140, 141]

### 3.1 Diffusion Enhancement

**FIGURE 3:**
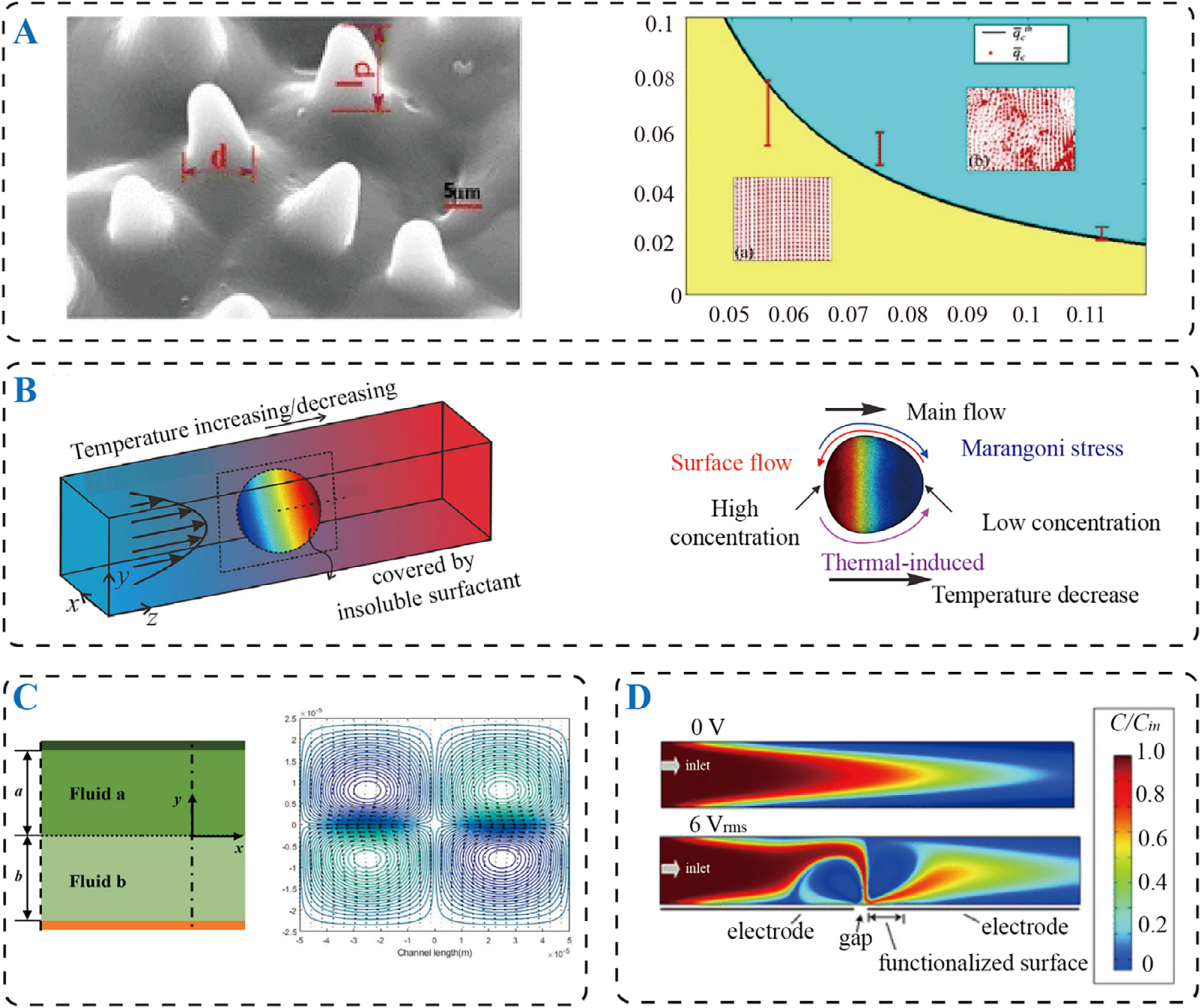
Diffusion enhancement in micro/nanostructures: (A) Schematic illustration of a biomimetic structure, showing the transition from Cassie‐Baxter state to Wenzel state occurs as the flow velocity increases [82]. Copyright 2017, Springer Nature. (B) Surface‐driven flow along a tangential temperature gradient [93]. Copyright 2020, AIP Publishing. (C) The motion and interfacial deformation of two separated fluids driven by thermocapillary effects [85]. Copyright 2024, Elsevier. (D) Localized vortices enhancing antigen transport and binding [94]. Copyright 2005, The Royal Society of Chemistry.

Enhancing molecular diffusion is a key strategy for improving mixing in micro/nanostructures operating under laminar flow, where convective effects remain limited by low *Re*. One effective approach is to engineer nanotextured surfaces on channel walls, which disrupt the near‐wall boundary layer and increase interfacial transport. Hierarchical structures that combine micro/nanoscale features perform better than single‐scale textures [81], as they generate multiscale flow perturbations. Biomimetic design has further broadened the possibilities of surface engineering, with natural structures inspiring new topographical patterns for efficient mixing. As shown in Figure 3A, a microchannel with a superhydrophobic wall inspired by lotus leaves exhibits stick‐slip motion at low flow velocities due to the Cassie‐Baxter state. When the flow exceeds a critical velocity, the surface can undergo a wetting transition from the Cassie–Baxter state to the Wenzel state. This transition perturbs the otherwise laminar flow near the biomimetic rough surface, thereby enhancing interfacial mixing [82, 83]. This bionic approach has also been applied to create rose‐petal‐like textures with high adhesion and anisotropic wettability on micromixer surfaces [84]. These structures disrupt surface tension, allowing the liquid to maintain flow anisotropy under low *Re*. The underlying mechanism is rooted in wetting and adhesion dynamics at the liquid‐solid interface. Hierarchical textures that preserve the Cassie‐Baxter state can sustain low‐friction slip flows and interfacial instabilities, which further promote mixing, particularly under laminar flow conditions [79, 80].

Beyond structural modification of solid boundaries, interfacial effects driven by external stimuli provide additional pathways to enhance diffusion‐dominated mixing. Thermocapillary effects also serve as an effective approach for enhancing diffusion‐driven mixing in micro/nanostructures and have been widely utilized for droplet transport [86, 87, 88, 89]. This mechanism relies on temperature gradients along fluid interfaces, which induce variations in surface tension and consequently generate fluid motion [90]. This resulting flow, driven by interfacial stress and deformation, is commonly referred to as the Marangoni effect [91, 92]. As illustrated in Figure 3B, temperature gradients within micro/nanostructures cause the liquid to migrate toward regions of lower temperature [90, 93]. Through the analysis of multilayer fluids in micro/nanostructures under thermocapillary effects, it is evident that thermal stimuli can readily disturb fluid interfaces, leading to interfacial deformation and the generation of recirculating flows around the interface [85]. These flow patterns promote both mixing and transport within micro/nanostructured systems. As illustrated in Figure 3C, the evolution of stacked fluid interfaces driven by thermocapillary forces highlights the strong potential of this mechanism for enhancing mixing efficiency.

In addition to thermally induced interfacial flows, electrically induced thermal effects offer another effective approach to introduce fluid motion and enhance mass transport. Electrothermal effects provide another important electrokinetic pathway for enhancing mixing, particularly in high‐conductivity fluids where conventional electroosmotic flow becomes less effective. Under an alternating electric field, Joule heating creates temperature gradients. These gradients trigger spatial variations in electrical conductivity and permittivity, thereby generating electrothermal body forces that drive fluid motion [48]. This mechanism introduces microscale convection into otherwise diffusion‐dominated systems, thereby significantly enhancing mass transport (as shown in Figure 3D). Optimized electrode layouts enable controllable cycling at low voltages, reduce thermal side effects, and achieve precise fluid mixing [95]. Both experimental measurements and numerical simulations have demonstrated that electrothermal flow is an energy‐efficient strategy that can produce characteristic velocities on the order of 100 µm s^−^^1^ with only a modest temperature increase (about 3 K) [94]. Overall, electrothermal actuation effectively breaks the constraint of purely diffusive transport in laminar microflows, enabling a transition toward convection–diffusion coupled mixing and substantially improving mixing efficiency in microfluidic systems.

### 3.2 Chaotic Advection

While diffusion enhancement strategies improve mixing by increasing interfacial transport and molecular exchange, their efficiency remains inherently limited by the rate of molecular diffusion. To overcome this limitation, micro/nanostructures that actively induce fluid stretching and folding through chaotic advection have been developed.

**FIGURE 4:**
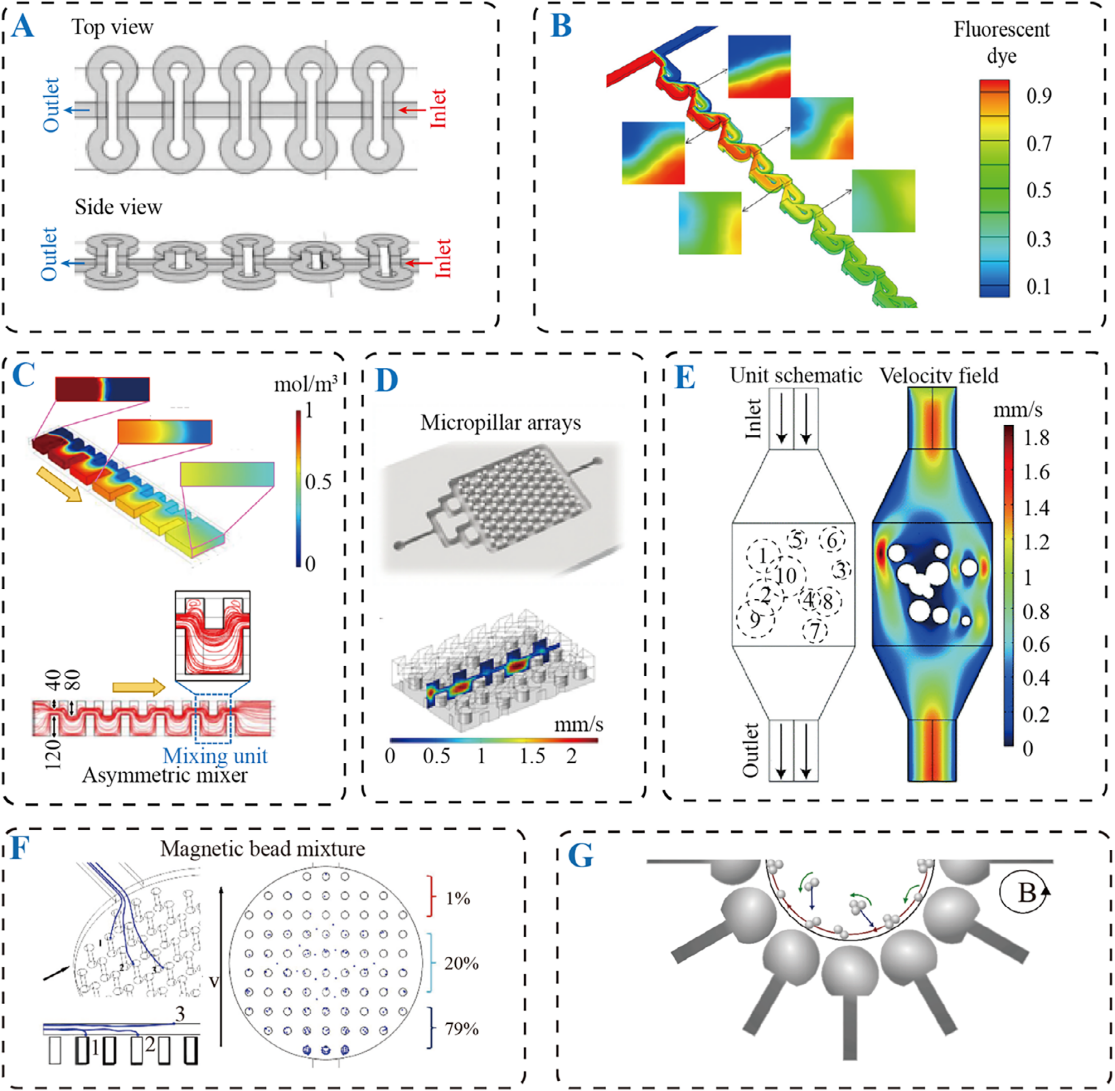
Chaotic advection generation in micro/nanostructures: (A) SAR design incorporating staggered herringbone grooves for rapid transverse dispersion [97]. Copyright 2021, Springer Nature. (B) The 3D SAR structure enables vertical and lateral redistribution of fluids [107]. Copyright 2015, Elsevier. (C) Asymmetric contraction‐expansion micromixer enhancing interfacial contact and fluid exchange via localized vortices [113]. Copyright 2022, MDPI. (D) Optimization of the arrangement, diameter, height, and spacing of microcolumns to achieve efficient fluid mixing [118]. Copyright 2025, Xin Shou et al. (CC BY 4.0). (E) Micropillar layouts optimized using genetic algorithms to improve mixing while reducing pressure drop [112]. Copyright 2019, The Royal Society of Chemistry. (F) Schematic illustration of local magnetic field gradients for liquid mixing [121]. Copyright 2020, Elsevier. (G) Liquid mixing driven by rotating magnetic field–controlled magnetic beads [119]. Copyright 2022, Springer Nature.

Chaotic advection is a key mechanism in micro/nanostructured fluidic mixing strategy that significantly enhances mixing efficiency. Recent studies, based on the principles of geometry‐induced mixing, split and recombine (SAR) structures have been continuously developed. SAR micro/nanostructures operate by repeatedly dividing fluid streams into smaller substreams and subsequently recombining them through spatially arranged channel geometries, which results in a higher pressure drop in the channel [98, 99]. Previous studies have proven that pressure gradients within parallel‐plane SAR geometries could drive vertical convection, further improving mixing rates [100]. This repetitive segmentation and rejoining process dramatically increases the interfacial area between fluids, promotes transverse dispersion, and enables effective chaotic advection even under low *Re* conditions [101, 102]. The results show that larger flow diameters combined with smaller curvature radii significantly increase the magnitude of Dean flow. This enhancement arises from centrifugal forces acting perpendicular to the main flow direction, which induce transverse and radial mixing [96]. Further extensions of the SAR concept include the incorporation of secondary patterns, such as staggered herringbone (SHB) grooves (shown in Figure 4A), which enable near‐instantaneous mixing with minimal sample consumption [97].

To further enhance mixing efficiency, researchers have proposed a SAR design featuring more complex 3D multilayer channel architectures, where multiple stacked or interleaved flow paths are introduced to increase the contact interfaces between fluid streams [104, 105, 106]. Compared with planar SAR structures, these multilayer architectures offer significant advantages. As shown in Figure 4B, their complex geometries enable both vertical and lateral redistribution of fluids within microchannels, resulting in more uniform mixing and accelerated diffusion [107, 108]. By integrating alternating flow redirection, significant enhancement in mixing efficiency can be achieved over a wide range of *Re* without requiring high shear forces, making it a versatile mixing strategy under diverse flow conditions [16, 109]. One of the classic examples of this 3D SAR design is the multi‐layer serpentine cross‐channel micro/nanostructure, which has been shown to achieve highly efficient mixing (>95%) across a relatively wide range of *Re* [110]. Additionally, this design can incorporate successive O‐type and X‐type segments, optimizing mixing at low flow rates over short distances. This arrangement achieves nearly perfect mixing, with efficiency approaching 99%, making it particularly useful for applications that require precise and rapid mixing at low sample volumes [111]. By reducing energy requirements while maintaining superior performance, 3D SAR structures have demonstrated great versatility for applications including diagnostics, biosensing, and drug delivery [103].

Beyond structured recombination strategies, asymmetric geometries provide an alternative route to induce chaotic advection through flow perturbation and symmetry breaking. Asymmetric obstacle arrangements are widely explored as passive strategies for inducing chaotic advection. By placing irregularly spaced or angled microstructures in the channel, these designs create complex flow perturbations that break streamline symmetry and promote repeated stretching and folding of fluids. As shown in Figure 4C, abrupt changes in channel cross‐section generate localized chaotic flows that enhance interfacial contact and mixing efficiency [113]. Asymmetric micro/nanostructures also include the incorporation of baffles, helical wires, and micropillars within fluidic channels. These designs induce abrupt changes in flow cross‐section and increase interfacial area, thereby enhancing mass transfer and improving mixing performance [63, 114, 115, 116]. Studies have demonstrated that obstacles and baffles arranged in the mixing chamber reduce mixing time about 54%–66%, highlighting the role of geometric perturbations in accelerating homogenization [117]. Taking micropillar integration as an example, studies have shown that higher pillar density can enhance mixing performance, while the system maintains reliable switching functionality over multiple operating cycles. As shown in Figure 4D, continuous optimization of micropillar arrangement, diameter, height, and spacing further expands their applicability [118]. Recently, studies have employed genetic algorithms to optimize micropillar geometries, significantly enhancing mixing efficiency in micro/nanostructures. As shown in Figure 4E, the algorithm‐generated pillar arrangement achieves maximal mixing performance with minimal pressure drop, thereby reducing energy loss [112].

In addition to passive geometric designs, active approaches based on external fields, such as magnetic actuation, have been introduced to further enhance chaotic mixing. Magnetic element mixing involves introducing micro/nanoscale magnetic beads into the fluid and using an external magnetic field to actively induce microscale perturbations that enhance mixing. Magnetic nanoparticles, typically 1–100 nm in size, combine general nanomaterial properties. Such as large surface area and quantum confinement effects, with unique magnetic characteristics, including superparamagnetic and strong magnetic responsiveness [120]. The efficiency of magnetic bead mixing depends largely on the design of the applied magnetic field. A common approach integrates soft magnets into PDMS microstructures to generate strong local magnetic field gradients, as shown in Figure 4F. Combining these fields with different structured channels can achieve diverse mixing behaviors and efficiencies [121]. Based on the effective chaotic advection induced by micropillar structures, a micro obstacle system integrating Nd‐Fe‐B material integrated with PDMS has been developed. When combined with magnetic beads in the fluid, this platform enables both efficient fluid mixing and sorting [122]. Moreover, mathematical algorithms can be employed to continuously optimize magnetic bead design, thereby improving fluid control precision and result reliability [123]. Several studies have also combined magnetic actuation with system‐level integration to create more efficient and compact micromixers. In Figure 4G, a planar rotating magnetic field is applied to a circular chamber. Reverse rotation between the chamber wall and central magnetic beads generates efficient mixing, and the mixing efficiency is primarily governed by the externally applied magnetic field [119].

### 3.3 Vortex Generation

Although chaotic advection significantly enhances mixing through repeated stretching and folding of fluid elements, the generation of well‐defined vortical structures provides an additional and often more intense mechanism for promoting convective transport. This has led to the development of vortex‐based mixing strategies in micro/nanostructures.

**FIGURE 5:**
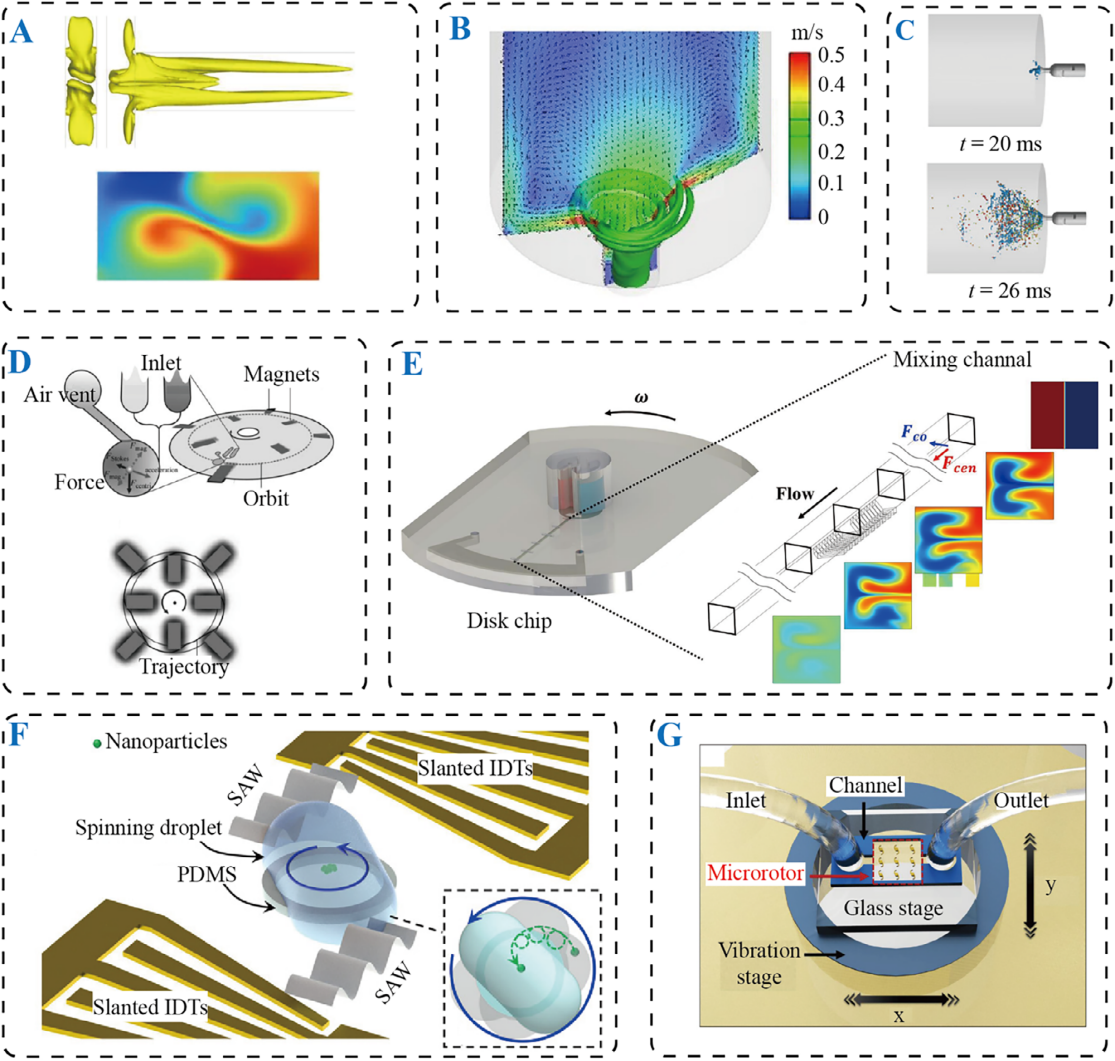
Vortex flow generation in micro/nanostructures: (A) Vortex formation in structured microchannels [127]. Copyright 2024, Elsevier. (B) Pressure‐induced high‐speed vortex mixing [129]. Copyright 2023, Elsevier. (C) High‐velocity flow leading to nozzle‐induced atomization [19]. Copyright 2020, Wiley. (D) Rotation‐enhanced mixing via magnetic actuation and disk oscillation [133]. Copyright 2005, The Royal Society of Chemistry. (E) Secondary vortices generated by herringbone grooves [130]. Copyright 2024, Elsevier. (F) SAW for vortexes generation of fluid [138]. Copyright 2021, The American Association for the Advancement of Science. (G) Acoustic streaming‐driven synchronized rotation [135]. Copyright 2020, Wiley.

Generating vortex flows in microstructures has therefore become a key strategy to enhance fluid mixing [124]. Contraction‐expansion arrays create abrupt changes in channel cross‐section, generating localized transverse vortices that disrupt laminar flow and promote fluid exchange and further boost convective mixing [125]. When *Re* > 500, vortices become unstable and shed asymmetrically in a periodic manner, known as vortex shedding [126]. Building on this principle, channels with circular barrier orifices can be designed using 3D printing technology. At the microscale, these structures generate dynamic vortices that achieve efficient liquid mixing, as shown in Figure 5A [127]. Another common design is the contraction‐expansion structure, which increases fluid pressure through sudden contraction, enabling mixing and subsequent atomization [128]. Based on these principles, the swirling flow reactor (SFR) has been developed, shown in Figure 5B. This SFR enhances liquid mixing and energy transfer through pressure‐driven swirling flows [129]. These structural designs have also inspired the development of various liquid nozzles. Specifically, as fluid velocity peaks near the nozzle outlet, it optimizes mixing efficiency and triggers the formation of multi‐scale vortices, as illustrated in Figure 5C [19].

Beyond these geometry‐induced vortex mechanisms, rotational platforms introduce additional body forces that can further intensify vortex formation. In centrifugal lab‐on‐a‐disk (LOD) platforms, vortex formation is further promoted by the combined effects of Coriolis, centrifugal, and Euler forces. Coriolis‐based mixing has been systematically studied in simple geometries such as straight or U‐shaped channels. Results show that combining rotation with structural modifications, including obstructions or width constrictions, can improve mixing by nearly 50% [131]. As shown in Figure 5D, U‐shaped channels on LODs generate Dean flows and Coriolis vortices, which significantly accelerate fluid homogenization. An operation mode based on magnetic beads and intermittent rotational cycling can reach 90% mixing of 25 µL samples in less than 1 s [132, 133]. Mixing efficiency is further enhanced when micro/nanostructures with nanotextured surface are rotated, since grooves patterned along the channel walls induce transverse secondary flows that generate steady or periodic vortices [134]. Numerical simulations confirm that introducing herringbone grooves into rotating microchannels synergizes with the Coriolis force, achieving over 90% mixing within just 20 mm of channel length, as shown in Figure 5E [130]. These findings highlight the effectiveness of combining curvature‐induced and surface‐pattern‐induced secondary flows for high‐performance mixing.

In addition to passive and rotation‐induced vortex generation, acoustic actuation provides a contactless and highly controllable approach to induce vortical flows. Surface acoustic wave (SAW) techniques provide a simple and versatile method for pump‐free manipulation of fluids by generating surface waves through resonant electrodes [136, 137]. As shown in Figure 5F, an acoustofluidic centrifuge driven by SAWs enables efficient droplet spinning and vortex generation in fluids [138]. By integrating vibrating elements, mechanical oscillations applied to channel microstructures generate shear forces and pressure fluctuations, disrupting laminar flow and significantly enhancing mixing performance [139]. A circular vibration system developed based on SAW technology, has also been developed to generate rotating microflows and capture single moving cells. When combined with a microcolumn array, this system supports parallel capture and motility assessment of large cells (>50 µm), overcoming limitations of traditional methods [140, 141]. Figure 5G illustrates a simple approach for uniformly actuating multiple microrotors fabricated by two‐photon polymerization. Using a piezoelectric vibration stage, acoustic streaming from curved sharp tips drives controllable rotor motion for microfluidic applications [135].

## 4 Biomedical Applications

### 4.1 Biomaterials Fabrication

**FIGURE 6:**
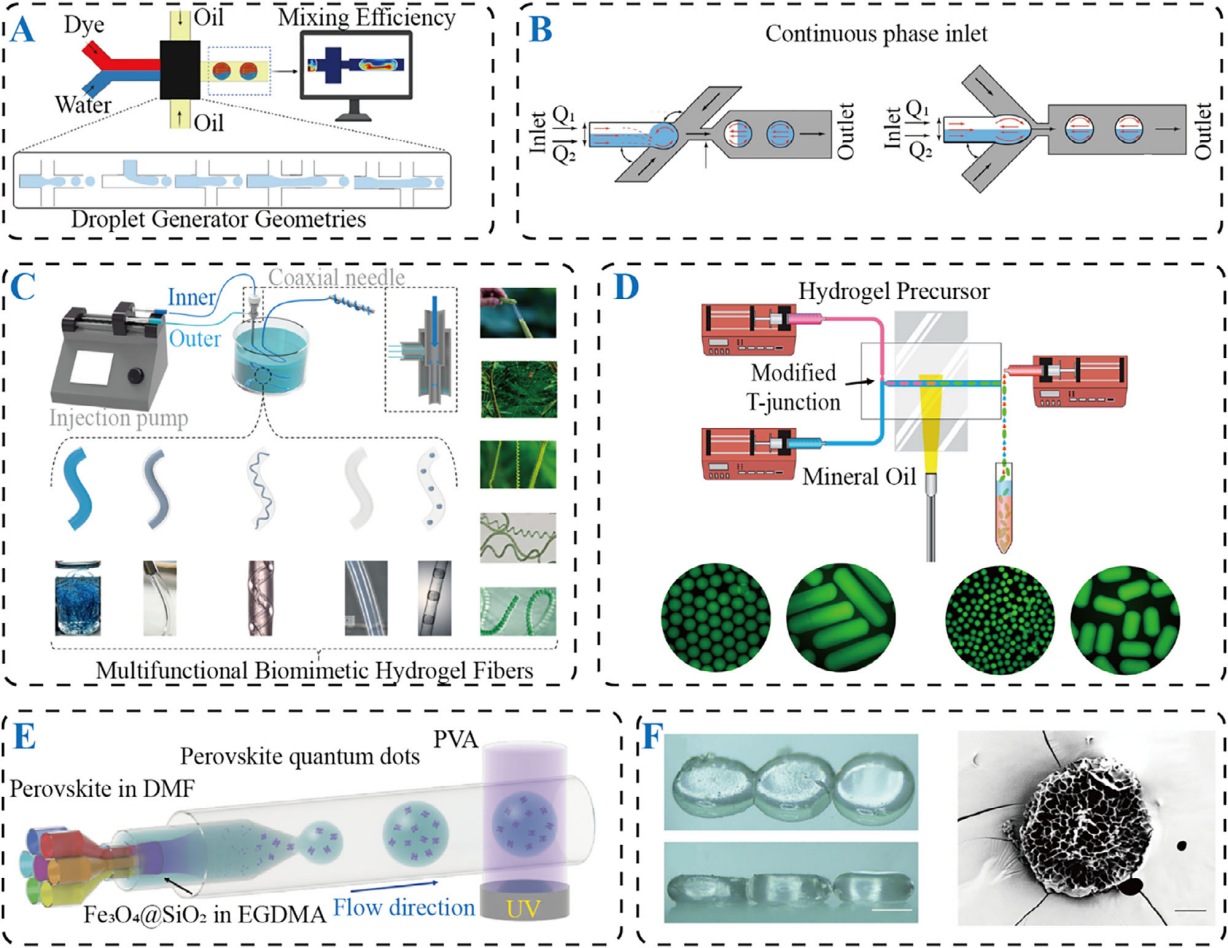
Representative micro/nanostructured fluidic mixing strategies for biomaterials fabrication: (A) Schematic of the cross‐T geometric structure [144]. Copyright 2024, Springer Nature. (B) Schematic of an asymmetric inlet layout that can precisely control droplet size [49]. Copyright 2021, Springer Nature. (C) Schematic of the structured co‐flow device producing multicore hydrogel fibers [150]. Copyright 2024, Mengwei Jia et al. (CC BY 4.0). (D) Modified T‐junction device through confined UV crosslinking of droplets [151]. Copyright 2020, American Chemical Society. (E) Liquid mixing microfluidics for biological coding [154]. Copyright 2021, Springer Nature. (F) Optical images and SEM images of the ELMP [44]. Copyright 2024, Elsevier.

Micro/nanostructured fluidic mixing technologies have demonstrated significant potential in the controllable fabrication of biomaterials. In particular, mixing strategies based on multiphase flow have emerged as a cornerstone for biomedical material fabrication. By confining and narrowing flow paths to induce intense velocity gradients, these methods facilitate the production of fibers and microspheres with precisely tunable morphologies, compositions, and functionalities [142, 143]. As illustrated in Figure 6A, through rational micro/nanostructure design, droplet size and morphology can be precisely controlled [144, 145]. Moreover, introducing asymmetry into micro/nanostructures has been shown to accelerate mixing up to six times compared with symmetric designs, highlighting the critical role of geometric imbalance in disrupting flow coherence (Figure 6B) [49].

In fiber fabrication, microfluidic spinning has emerged as a versatile strategy to produce continuous hydrogel or polymeric fibers with adjustable dimensions and internal architectures [146]. Typically relying on coaxial flow designs, these platforms enable laminar sheath‐core stream interactions, followed by fiber solidification through ionic crosslinking, photopolymerization, or solvent exchange. Alginate and GelMA‐based microfluidic spinning systems have been widely applied to fabricate cell‐laden scaffolds for tissue engineering [147]. Beyond simple geometries, the integration of grooves, ridges, or patterned channels can harness secondary flows and controlled stream deformation. This enables the bionic fabrication of twisted, helical, or multilayered fibers that closely recapitulate complex native tissue architectures [148, 149]. As shown in Figure 6C, diverse microfiber structures fabricated through microfluidics can encapsulate multiple cell types or construct vascularized and immune‐responsive scaffolds [150]. Furthermore, microtubes produced by frequency modulated pulsatile flows exhibit superior liquid diversion performance, opening new avenues for the design of advanced fluidic circuits [38].

In addition to fibers, microfluidic technologies have provided precise control over droplet dynamics and interfacial processes, enabling the synthesis of monodisperse hydrogel microspheres with customized structures and properties. As shown in Figure 6D, droplets subjected to ultraviolet crosslinking within confined microchannels of a modified T‐junction can yield microspheres with varied morphologies [151]. Furthermore, with the development of multiphase microsphere fabrication strategies, variations in capillary microchannel configurations allow fluids to autonomously execute complex mixing algorithms based on capillary‐driven flow timing [152]. Building upon programmable microfluidics, fluidic mixing technologies have further advanced toward applications in biological information encoding and information security, as illustrated in Figure 6E [153, 154]. At present, functional microcarriers fabricated via micro/nanostructures have been widely applied in biomedical fields. A yeast‐inspired antioxidant porous microcarrier exhibited excellent biocompatibility and high porosity, making it well‐suited for drug delivery and cell therapy applications [155]. More recently, biomimetic erythrocyte‐like microparticles (ELMPs) were developed as efficient scaffolds for cell adhesion while simultaneously exhibiting oxygen transport capacity and near‐infrared‐responsive release of growth factors, highlighting their promise for regenerative medicine (Figure 6F) [44].

In parallel with these passive and structure‐assisted strategies, electrokinetic mixing has emerged as a complementary active approach for multiphase manipulation. By introducing electrospray on micro/nanostructured surfaces, interfacial instabilities and fine droplet breakup can be induced, enabling rapid mixing and precise control over fluid interfaces [156]. The mixing behavior can be readily tuned by adjusting the applied DC voltage, offering a high degree of controllability over droplet formation and internal mixing states. Compared with purely geometry‐dependent methods, this electrically driven strategy decouples mixing efficiency from complex structural designs. By reducing reliance on intricate architectures while maintaining high performance, this approach streamlines device fabrication and enhances the versatility of microfluidic platforms [44, 157]. In these systems, efficient mixing within droplets is essential for achieving uniform solute distribution and consistent particle properties, underscoring the critical role of micro/nanostructured fluidic mixing in high‐fidelity biomaterial fabrication.

### 4.2 Drug Development

**FIGURE 7:**
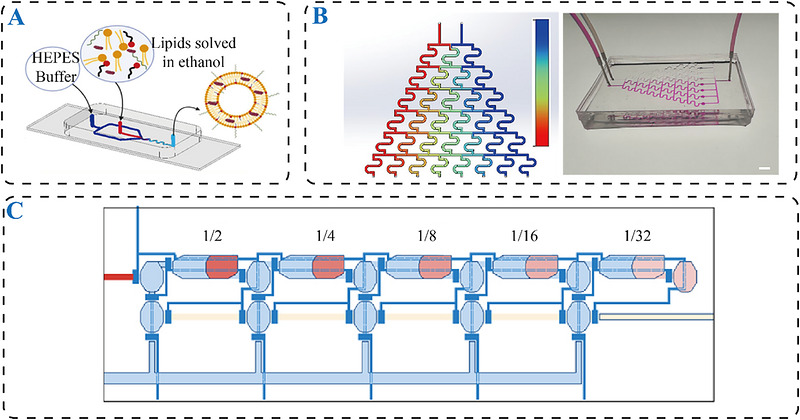
Representative micro/nanostructured fluidic mixing strategies for drug development: (A) High‐throughput droplet microfluidic chip generating PEGylated liposomes [158]. Copyright 2024, Elsevier. (B) Schematic of a microfluidic platform capable of constructing eight independent drug concentrations in parallel [160]. Copyright 2025, Wiley. (C) Gradient‐generating microfluidic chip enabling continuous drug dilution [162]. Copyright 2019, AIP Publishing.

Micro/nanostructured mixing technologies have become indispensable in drug development. By facilitating enhanced mass transport and reaction uniformity, these strategies minimize reagent consumption and accelerate the transition from fundamental research to clinical translation. In drug nanocarrier fabrication, precise control over nanoparticle size, monodispersity, and encapsulation efficiency can be achieved through the deliberate tuning of micro/nanostructural dimensions. For example, a high‐throughput microfluidic strategy has been demonstrated for scalable synthesis of PEGylated liposomes with tunable sizes (60‐150 nm, PDI < 0.2) through optimization of chip geometry, flow conditions, and lipid composition (Figure 7A) [158]. To better replicate physiological microenvironments, microfluidic platforms have been developed for 3D cell culture and drug response assessment. These systems enable spheroid formation and long‐term culture under stable drug gradients, yielding responses more reflective of in vivo conditions [159]. As shown in Figure 7B, a microfluidic array comprising eight parallel microchannels establishes discrete concentration gradients via hydrodynamic flow focusing, providing an effective bridge between conventional in vitro assays and in vivo studies [160]. Beyond efficacy testing, micro/nanostructured microfluidics have been extended to drug metabolism studies. By integrating intestinal, hepatic, skin, and renal analogs, multi‐organ microphysiological systems recapitulate complex ADME (absorption, distribution, metabolism, and excretion) processes, while facilitating high‐throughput drug efficacy evaluation with minimal reagent consumption [161]. Continuous‐flow microfluidic devices remain widely employed for drug screening due to their straightforward implementation and ability to sustain uninterrupted flow driven by mechanical pumps or electrokinetic forces. As shown in Figure 7C, microfluidic chips equipped with serial dilution generators can establish linear or logarithmic drug concentration gradients for cytotoxicity testing on cancer cells such as HepG2 [162]. Collectively, the integration of precise micro/nanostructured fluidic mixing with gradient generation, dilution, and 3D cell culture within continuous‐flow, droplet‐based, and digital microfluidic platforms highlights their transformative potential in advancing personalized drug development, toxicity screening, and precision medicine.

### 4.3 Cell Culture and Organs‐on‐Chips

**FIGURE 8:**
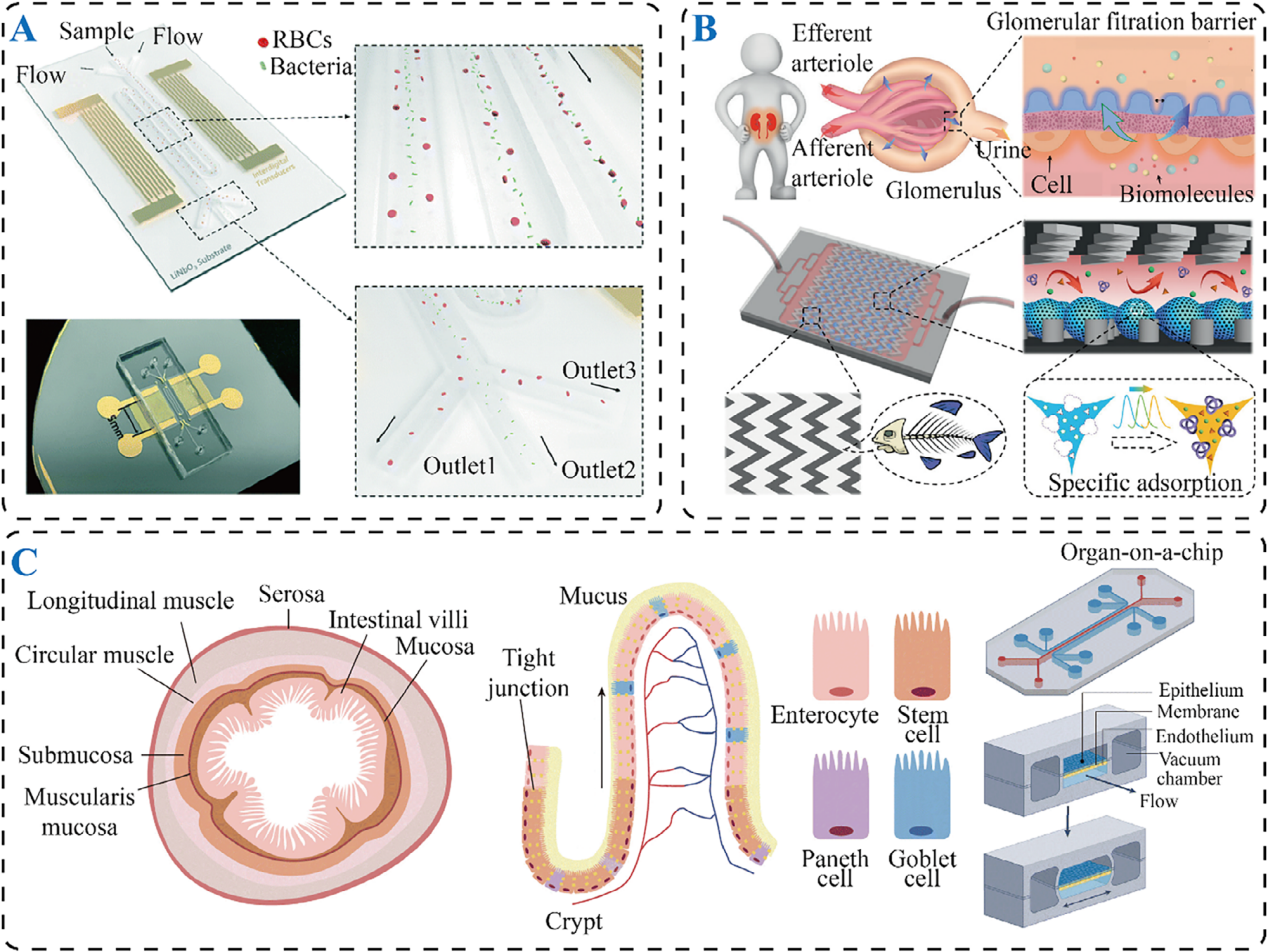
Representative micro/nanostructured fluidic mixing strategies for advanced cell culture and organ‐on‐a‐chip systems: (A) Schematic of the serpentine microchannel coupled with acoustic wave actuation [165]. Copyright 2021, The Royal Society of Chemistry. (B) Mechanism of the MIPIOPs embedded within a herringbone microfluidic chip [168]. Copyright 2021, Wiley. (C) Schematic of gut‐on‐a‐chip system with physiologically relevant flow and architecture [169]. Copyright 2025, The Royal Society of Chemistry.

In the design of advanced in vitro cell culture platforms and organ‐on‐a‐chip systems, precise regulation of nutrient delivery, and the cellular microenvironment is critical for faithfully recapitulating physiological conditions [163, 164]. For instance, integrating serpentine microstructures with acoustic waves, high cell recovery and viability are maintained, while ensuring a stable recovery process and achieving high‐throughput performance (Figure 8A) [165]. When combined with microcolumn array modules, such platforms enable quantitative assessment of cytoskeletal dynamics, nuclear deformation, and traction forces across substrates of varying stiffness and geometries, thereby offering more physiologically relevant environments for cells [166, 167]. These capabilities further allow label‐free, real‐time interrogation of cell‐substrate interactions, which are central to studies of stem cell differentiation, mechanotransduction, and cancer invasion. Micropillar arrays, serving as controlled microscale obstacle fields, provide additional opportunities to study cellular motility and collective behavior under well‐defined physical constraints.

Organs‐on‐chips have witnessed remarkable progress over the past decade, emerging as transformative tools to replace conventional cell culture and animal models owing to their miniaturization, integration, physiological relevance, and biomimetic microenvironments. With parallel advances in materials science and computational biology, diverse platforms such as heart‐, lung‐, and brain‐on‐chips have been realized, and recent incorporation of machine learning has further enhanced data analysis and predictive capacity [39]. As illustrated in Figure 8B, a blood purification platform combining molecularly imprinted inverse opal particles with a herringbone microfluidic chip achieved high adsorption efficiency, mimicking kidney‐like filtration functions [168]. Similarly, intestine‐on‐chip models have successfully reconstructed barrier functionality by integrating 3D villus‐like microstructures and reproducing intestinal shear stress conditions, as shown in Figure 8C [169]. Collectively, these platforms have become powerful tools for regenerative medicine, disease modeling, and patient‐specific drug testing, underscoring the indispensable role of micro/nanostructured fluidic mixing in next‐generation biomedical systems.

### 4.4 Biosensing and Diagnostics

Micro/nanostructured fluidic mixing technologies have become essential, enabling significant advances in biosensing and diagnostic platforms. In clinical settings, the rapid and sensitive detection of proteins, nucleic acids, metabolites, and cells is critical for early disease diagnosis, treatment response monitoring, and personalized therapeutic strategies. When integrated with microfluidic systems, these approaches enable miniaturized, automated, and low‐reagent‐consumption platforms for point‐of‐care testing (POCT). Such technologies enable the development of high‐performance biosensors that can be deployed in clinical, home, and field environments [170].

**FIGURE 9:**
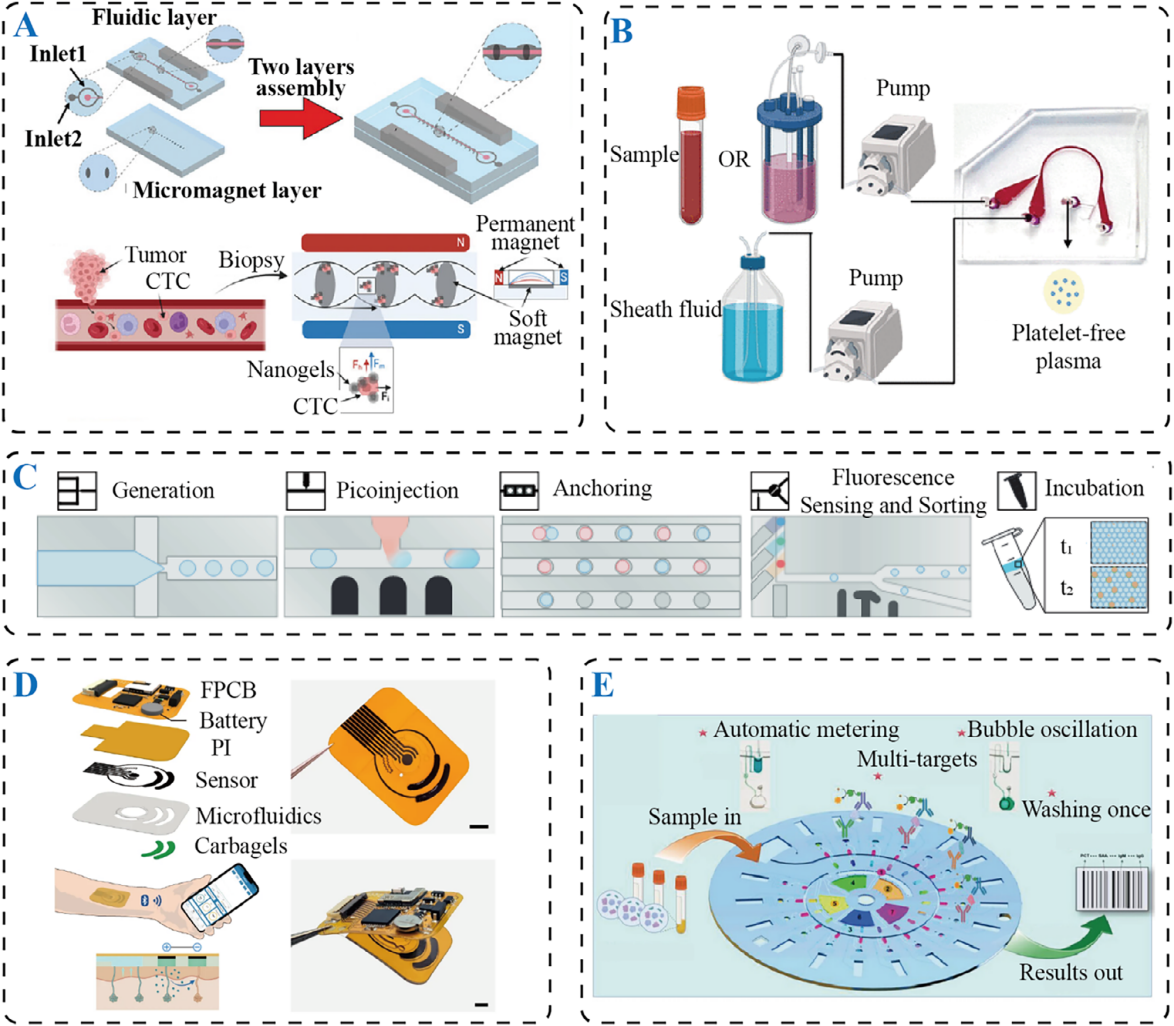
Representative micro/nanostructured fluidic mixing strategies for biosensing: (A) Schematic of dual‐layer magnetic microfluidic chip integrating thermoresponsive fluorescent nanogels [171]. Copyright 2023, Wiley. (B) Schematic of ExoArc platform combining SEC and flow‐guided microchannel [175]. Copyright 2024, American Chemical Society. (C) Schematic of programmable and reconfigurable microfluidic systems [177]. Copyright 2025, Springer Nature. (D) Schematic of a wearable patch for CRP POCT [180]. Copyright 2023, Springer Nature. (E) Schematic of the “Dac” chip with siphon valves and micromixers [181]. Copyright 2024, Wiley.

In cancer liquid biopsy, micro/nanostructured microfluidics has achieved unprecedented sensitivity and multiplexing capacity, facilitating early diagnosis, prognosis, and therapeutic monitoring. For instance, a dual‐layer magnetic microfluidic device coupled with temperature‐responsive fluorescent nanogels enables the efficient capture and in situ staining of circulating tumor cells (CTCs) [171] (Figure 9A). To detect circulating tumor DNA (ctDNA), the IC3D platform integrates droplet generation with fluorescent PCR and 3D particle counting, achieving a remarkable 0.00125% detection limit for KRAS G12D mutations with zero false positives [172]. Similarly, enzyme‐free droplet assays based on CHA, such as ddaCHA, have achieved a sensitivity of 10 fM for room‐temperature miRNA detection [173, 174]. Beyond nucleic acids, microfluidic systems have been optimized for the high‐purity enrichment of exosomes via size‐exclusion chromatography (SEC), effectively eliminating platelet contamination [175] (Figure 9B). Furthermore, platforms like DMF‐Protein‐seq and shear‐flow chambers coupled with single‐cell mass spectrometry allow for the profiling of proteins and metabolites, revealing tumor heterogeneity and metabolic divergence among CTC subpopulations [176]. Finally, droplet microfluidic component libraries (Figure 9C) offer a versatile, cost‐effective framework for assembling multi‐step diagnostic workflows, such as biological coding and information exchange [177].

For POCT, micro/nanostructured fluidic mixing technologies enable rapid, sensitive, and convenient diagnostics to be performed outside traditional laboratory settings. Microfluidic biosensing platforms allow point‐of‐care detection of essential inflammatory biomarkers such as C‐reactive protein (CRP), procalcitonin (PCT), and interleukin‐6 (IL‐6). A notable example is a 3D printed‐based microfluidic chip capable of simultaneously quantifying CRP, PCT, and IL‐6 via chemiluminescent immunoassays, achieving clinically relevant dynamic ranges with strong correlation to ELISA results [178]. Complementarily, a wearable NFC‐based electrochemical biosensor with nanobody‐modified electrodes enabled high‐sensitivity CRP monitoring without requiring sample pretreatment [179]. Moreover, a wearable patch integrating a sweat‐extraction microchannel with a graphene sensor array (Figure 9D) demonstrated real‐time CRP detection, with clinical validation confirming correlation to serum CRP levels in patients with heart failure and infectious diseases [180]. In addition, POCT platforms have provided efficient solutions for scalable diagnostics. As shown in Figure 9E, a high‐throughput “Dac” system combining a dual siphon valve and microbubble drive performed 17 enzyme‐linked immunosorbent assay (ELISA) reactions simultaneously, with minimal reagent consumption and high analytical sensitivity [181]. The POCT systems also have enabled rapid and decentralized biomarker quantification, supporting early diagnosis and timely intervention. For example, a microchip‐based liquid chromatography (LC) platform measured HbA1c levels within 2 min, offering a low‐cost and portable diagnostic option for diabetes [182]. In addition, the integration of smart devices enables remote monitoring of various indicators. For example, an oxygen‐sensitive microfluidic platform connected to a smart device allows low‐cost and portable quantitative detection of multiple chronic disease biomarkers, including bilirubin and creatinine [183]. These advances highlight the essential role of micro/nanostructured fluidic mixing strategies in shaping the next generation of POCT systems. By enabling rapid, accurate, and decentralized diagnostics across diverse medical conditions, these biosensing technologies are poised to accelerate personalized medicine, strengthen public health surveillance, and advance global healthcare equity.

## 5 Conclusion

In recent years, micro/nanostructured fluid mixing technologies have achieved remarkable progress. Initially developed to miniaturize chemical reactions, these systems have gradually evolved toward more sophisticated applications, including the realization of complex biological platforms such as organs‐on‐chips. A growing body of research has significantly expanded their applicability in the biomedical field. In this review, a novel classification framework is proposed to systematically summarize the development of micro/nanostructured fluidic mixing strategies. First, the fundamental principles governing micro/nanostructure design for fluid mixing are discussed, including both the theoretical basis of microscale fluid dynamics and the indispensable role of numerical simulation in guiding structural optimization. Subsequently, mixing strategies are categorized according to the dominant physical mechanisms involved, namely diffusion enhancement, chaotic advection, and vortex flow generation. These mechanisms span a spectrum from mild to intense mixing effects, highlighting not only the versatility and practical value of micro/nanostructures, but also providing a useful guideline for researchers to rationally select appropriate strategies based on specific application requirements. Furthermore, the biomedical applications of these mixing strategies are comprehensively reviewed, including their roles in biomaterial fabrication, drug development, cell culture, organs‐on‐chips platforms, as well as miniaturized biosensing and diagnostic systems. Despite these significant advances, several critical challenges remain to be addressed before the full potential of these technologies can be realized.

From a technical perspective, the choice of fabrication materials for micro/nanostructures still requires further development. Current approaches largely rely on PDMS, which imposes limitations in terms of scalability and structural complexity. Recent advances in nanotechnology and rationally designed biomaterials have demonstrated promising capabilities in dynamically regulating cellular functions and enhancing tissue regeneration [184, 185, 186, 187], which may provide new directions for the development of improved materials and structural designs. From the perspective of clinical translation, the transition from laboratory research to practical application remains at an early stage. Challenges such as the complexity of microfluidic integration, limitations in fabrication techniques, and high production costs continue to hinder large‐scale clinical adoption. In addition, structural design requires more comprehensive consideration to achieve highly efficient and controllable fluid mixing. Continuous optimization of geometric parameters and mixing processes is essential. Although specific designs, such as Tesla valve‐inspired geometries, have demonstrated advantages in regulating fluid residence time [188], expanding their applicability across diverse scenarios remains a significant challenge.

Addressing these issues requires deeper interdisciplinary integration across fields such as biology, materials science, and bioinformatics. In this context, the rapid advancement of AI provides new opportunities. The incorporation of machine learning and data‐driven approaches has shown great potential in accelerating structural design and optimizing mixing performance. For instance, AI‐assisted design frameworks can rapidly generate efficient mixing configurations tailored to specific experimental requirements, significantly reducing reliance on empirical trial‐and‐error methods. In particular, the further development and adoption of generative AI techniques are expected to enable automated and intelligent optimization of mixing strategies, providing more robust and reliable technical support for next‐generation microfluidic systems [189].

Looking forward, future research should focus on improving cell culture and induction methodologies, developing novel biomaterials with enhanced biocompatibility and functional fidelity, simplifying operational procedures, and strengthening interdisciplinary collaboration. A deeper understanding of fluid mixing behavior at the micro/nanoscale remains essential for elucidating fundamental mechanisms and driving technological innovation. Although significant challenges persist, continued innovation, effective integration of emerging technologies, and cross‐disciplinary efforts are expected to facilitate the successful translation of micro/nanostructured fluidic mixing systems into clinical and industrial applications. Ultimately, these advancements will contribute to the development of personalized medicine and provide more effective therapeutic solutions for a wide range of diseases.

## Conflicts of Interest

The authors declare no conflict of interest.

## Data Availability

The data that support the findings of this study are available in the public domain. These data were derived from the following resources available in the public domain: PubMed (PMID: 41661604) at https://pubmed.ncbi.nlm.nih.gov/41661604/.Data sharing is not applicable to this review article as no new data were generated or analyzed in this study.
